# Nonintubated video-assisted thoracoscopic surgery using adaptive servo ventilation in a patient with severe respiratory dysfunction: a case report

**DOI:** 10.1186/s40981-019-0278-2

**Published:** 2019-09-03

**Authors:** Yuki Kikuchi, Masaki Orihara, Rie Mieda, Shigeru Saito

**Affiliations:** 1 Department of Anesthesiology, Saiseikai Utsunomiya Hospital, 911-1 Takebayashi-machi, Utsunomiya-shi, Tochigi, 321-0974 Japan; 2 Department of Anesthesiology, Gunma University Graduate School of Medicine, 3-39-22 Showa-machi, Maebash-shi, Gunma 371-8511 Japan

**Keywords:** Nonintubated video-assisted thoracoscopic surgery, Adaptive servo ventilation, Respiratory dysfunction, Epidural anesthesia, Dexmedetomidine

## Abstract

**Background:**

Video-assisted thoracoscopic surgery (VATS) is usually performed under general anesthesia with a double-lumen tube. Recently, VATS without tracheal intubation in a patient with severe respiratory dysfunction has been reported. A case of nonintubated (also known as awake or tubeless) VATS using adaptive servo ventilation (ASV), a form of noninvasive positive pressure ventilation providing varying amounts of ventilator support, is presented. This is the first report of nonintubated VATS using ASV.

**Case presentation:**

A 60-year-old woman was scheduled for VATS bullectomy for the treatment of pneumothorax. She had severe respiratory dysfunction and had been receiving ASV therapy because of type 2 respiratory failure. Thus, nonintubated VATS using ASV, epidural anesthesia, and dexmedetomidine were selected. When surgical pneumothorax was created by incision of the pleura, her respiratory status remained stable. In addition, lung collapse was easily induced at operation. The leaking bulla was easy to identify, and bullectomy was performed. During surgery, she continued spontaneous breathing and did not complain of pain or discomfort. She was transferred to the intensive care unit with ASV and discharged on postoperative day 12 with no respiratory complications.

**Conclusion:**

It is necessary to maintain a stable respiratory status, as well as adequate analgesia and sedation, during nonintubated VATS in patients with severe respiratory dysfunction. When total lung collapse is not necessary for the surgical procedure, use of ASV would be an effective strategy.

## Background

Video-assisted thoracoscopic surgery (VATS) is usually performed under general anesthesia with a double-lumen tube to achieve single-lung ventilation [1]. However, general anesthesia and tracheal intubation carry high risks for patients with various thoracic diseases, including those with respiratory dysfunction. Recently, VATS without tracheal intubation has been reported to be feasible and safe for patients with respiratory dysfunction [1–5]. VATS without tracheal intubation is distinct from conventional general anesthesia with tracheal intubation because patients breathe spontaneously. However, spontaneously breathing patients’ oxygenation and ventilation can be impaired because of a surgical pneumothorax [2]. Therefore, careful respiratory management is essential to maintain the physiological status during VATS without tracheal intubation.

Adaptive servo ventilation (ASV) is a form of noninvasive positive pressure ventilation (NPPV) [6]. It provides a background level of expiratory positive airway pressure (EPAP) to which a variable amount of inspiratory pressure support (IPAP) is added. The pressure support (IPAP-EPAP) applied varies depending on the subjects’ ventilatory effort [7].

A case of nonintubated (also known as awake or tubeless) VATS bullectomy for the treatment of pneumothorax using ASV, epidural anesthesia, and dexmedetomidine in a patient with severe respiratory dysfunction is presented. This is the first report of nonintubated VATS using ASV.

## Case presentation

**Fig. 1:**
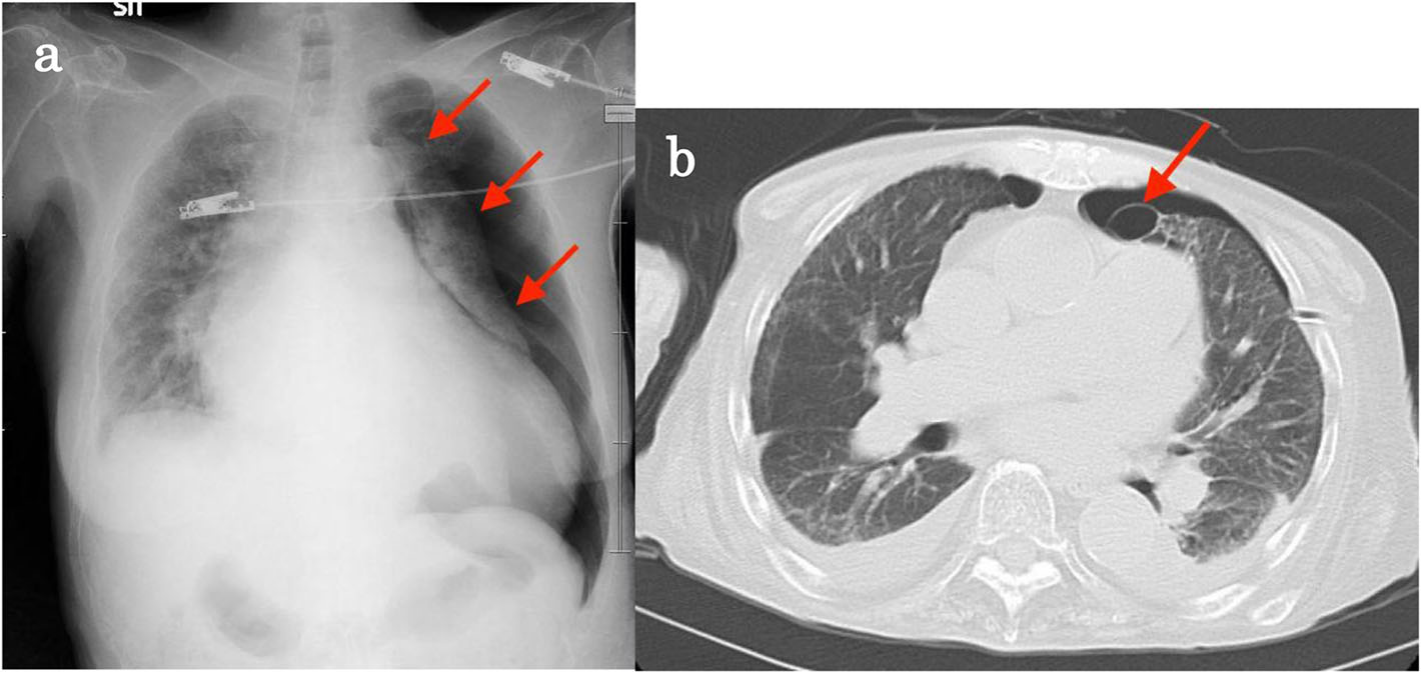
Preoperative images of the patient. **a** Chest X-ray shows the collapsed lung (red arrows). The left pneumothorax is severe. **b** Computed tomography shows a bulla of the left upper lobe behind the sternum (red arrow). The bulla was suspected as the cause of the pneumothorax.

A 60-year-old woman (height 154 cm, weight 35 kg) was transported to our hospital by ambulance with chest pain and dyspnea. After evaluation, she was diagnosed with left pneumothorax (Fig. 1). She had a history of muscular dystrophy, dilated cardiomyopathy, and atrial fibrillation. She had been receiving ASV therapy for 2 years because of type 2 respiratory failure. Before admission, she was classified as Hugh-Jones level 5 with the oxygen flow rate, IPAP, and EPAP set to 1 L/min, 7–16 cm H_2_O, and 4 cm H_2_O, respectively. Prehospital respiratory function test and transthoracic echocardiography results are presented in Table 1. Despite chest tube drainage and pleurodesis, the air leak persisted. Thus, VATS bullectomy was scheduled. It was thought that the operation would not take much time because of the substernal location of the bulla suspected as the cause of the pneumothorax. Moreover, she had severe respiratory dysfunction and refused tracheal intubation due to the risk of tracheal intubation, and she also considered that initiation of mechanical ventilation would make it difficult to wean from mechanical ventilation. Therefore, VATS without tracheal intubation was chosen.

**Table 1:** Prehospital respiratory function test and transthoracic echocardiography results

Respiratory function test	
Vital capacity (L)	0.89
% vital capacity (%)	32.80
Forced expiratory volume in one second (L)	0.82
Forced expiratory volume % in one second (%)	93.18
Transthoracic echocardiography	
Left ventricular end-diastolic diameter (mm)	47
Left ventricular end-systolic diameter (mm)	40
Ejection fraction (%)	35
Tricuspid regurgitation	Moderate
Mitral regurgitation	Mild

Standard monitoring included electrocardiogram, arterial blood pressure, and pulse oximetry. She was kept on ASV performed with a full face mask in the operating room. The settings were oxygen flow rate, 4 L/min; IPAP, 7–16 cm H_2_O; and EPAP, 4 cm H_2_O. After peripheral arterial cannulation, an epidural catheter was placed at the thoracic 5–6 level, and 3 ml of 0.5% ropivacaine were injected through the epidural catheter following 2 ml of 1% mepivacaine as a test dose. Then, an infusion of 1 μg/kg/h dexmedetomidine was started. Before she was turned to the right lateral position, it was confirmed that the extent of epidural anesthesia was from thoracic 2 to 8. Local anesthesia was not performed. A 5-cm skin incision was made in the third intercostal space. When surgical pneumothorax was created by incision of the pleura, her respiratory status remained stable. In addition, lung collapse was obtained with ease at operation while she was on ASV, a form of NPPV. In the middle of the operation, the dosage of dexmedetomidine was reduced to 0.6 μg/kg/h to maintain the Richmond Agitation-Sedation Scale around − 2. The leaking bulla was easily identified, and bullectomy was performed. After a negative air leak test, a chest tube was inserted. The operation time was 56 min, and the anesthesia time was 116 min. During surgery, although her arterial blood gas analysis showed respiratory acidosis (Table 2), oxygen saturation was maintained higher than 90%. In addition, she continued spontaneous breathing and did not complain of pain or discomfort. She was transferred to the intensive care unit (ICU) with ASV. The chest tube was removed on postoperative day (POD) 1, and she was transferred out of the ICU on POD 2. She was discharged on POD 12 with no respiratory complications.

**Table 2:** Preoperative and intraoperative arterial blood gas data

	Preoperation	At the time of operation	End of operation
pH	7.421	7.366	7.386
PaCO_2_ (mmHg)	45.3	53.4	50.8
PaO_2_ (mmHg)	123.0	125.0	154.5
Base excess (mEq/L)	4.6	4.8	5.0

## Discussion

A case of nonintubated VATS using ASV was described. Our experience indicates that ASV can be successfully used to maintain a stable respiratory status during nonintubated VATS with severe respiratory dysfunction, in combination with epidural anesthesia and dexmedetomidine.

Nonintubated VATS was first reported for diagnostic procedures in 1979 [5]. In recent years, nonintubated VATS strategies are becoming increasingly used worldwide [4]. However, there is always the possibility of the need to convert to general anesthesia with tracheal intubation, although complications and adverse effects following general anesthesia, tracheal intubation, and one-lung ventilation are inevitable. To prevent conversion to general anesthesia, stable spontaneous breathing following adequate analgesia and sedation is required [3].

In the present case, the patient had used ASV before admission. Thus, it was decided that the patient would continue on ASV in the operating room. Because ASV is a form of NPPV, the operated lung did not collapse completely during the surgical pneumothorax. With surgically induced open pneumothorax, the operated lung usually collapses progressively. The dependent lung is then responsible for sufficient respiratory function, including oxygenation and ventilation [2]. That would not allow the patient with severe respiratory dysfunction to achieve stable respiration. The present patient did not need total lung collapse because of the substernal location of the bulla suspected as the cause of her pneumothorax. If total lung collapse is not necessary, use of ASV would be an effective strategy during nonintubated VATS.

It is essential to provide adequate analgesia and sedation in addition to careful respiratory management. Various approaches to analgesia have been developed and proven feasible, including the current mainstream of thoracic epidural anesthesia, paravertebral nerve block, and percutaneous or thoracoscopic intercostal nerve block [8]. Moreover, various drugs such as propofol, midazolam, and dexmedetomidine have been used as sedatives [3, 8]. Dexmedetomidine provides an anxiolytic effect and cooperative sedation without respiratory depression [9]. Thus, dexmedetomidine may be a suitable sedative for nonintubated VATS.

## Conclusion

It is necessary to maintain a stable respiratory status during nonintubated VATS in patients with severe respiratory dysfunction. When total lung collapse is not necessary for the surgical procedure, use of ASV would be an effective strategy.

## Data Availability

Data relevant to this case report are not available for public access because of patient privacy concerns but are available from the corresponding author on reasonable request.

## Abbreviations

ASV: Adaptive servo ventilation
EPAP: Expiratory positive airway pressure
ICU: Intensive care unit
IPAP: Inspiratory positive airway pressure
NPPV: Noninvasive positive pressure ventilation
POD: Postoperative day
VATS: Video-assisted thoracoscopic surgery
